# Multisystemic sarcoidosis revealed by a Heerfordt syndrome: case report

**DOI:** 10.11604/pamj.2022.42.159.34073

**Published:** 2022-06-29

**Authors:** Glad Smart Moussounda Mpika, Paulvon Phérol Koumeka, Lamyae Amro

**Affiliations:** 1 Pneumology Department, Mohammed VI University Hospital, Faculty of Medicine and Pharmacy of Marrakech, Cadi Ayyad University, Marrakech, Morocco

**Keywords:** Granulomatosis, anterior uveitis, parotiomegaly, facial paralysis, case report

## Abstract

Heerfordt syndrome is a rare clinical form of sarcoidosis with a favorable evolutionary profile in the majority of cases. In its classical form, it associates uveitis, parotidomegaly, facial paralysis and fever. We report a case of multisystemic sarcoidosis type II revealed by a Heerfordt syndrome in a 51-year-old female patient.

## Introduction

Sarcoidosis is the most common systemic granulomatosis and diffuses infiltrative disease of unknown etiology. It can affect the whole body with pulmonary and lymphatic tropism [1,2]. In 1909, Heerfordt described a syndromic entity associating uveitis, parotitis and facial paralysis in a febrile context [3,4]. Waldenstrom in 1937 had linked this syndrome to sarcoidosis, a rare manifestation of the disease which is easily diagnosed in its complete form [5]. We report a case of Heerfordt syndrome revealing a multisystem sarcoidosis.

## Patient and observation

**Patient information:** a 51-year-old woman, housewife, exposed to wood smoke, followed by a visceral surgery for a lithiasis gallbladder and referred to a pneumology consultation in front of a pathological thoracic imaging preceded by a symptomatology evolving for seven months made of a dry cough, a dyspnea stage II of Sadoul associated with a decrease of the bilateral visual acuity with xerophthalmia, xerostomia as well as bilateral polyartralgias of the big joints (knees and ankles).

**Figure 1:**
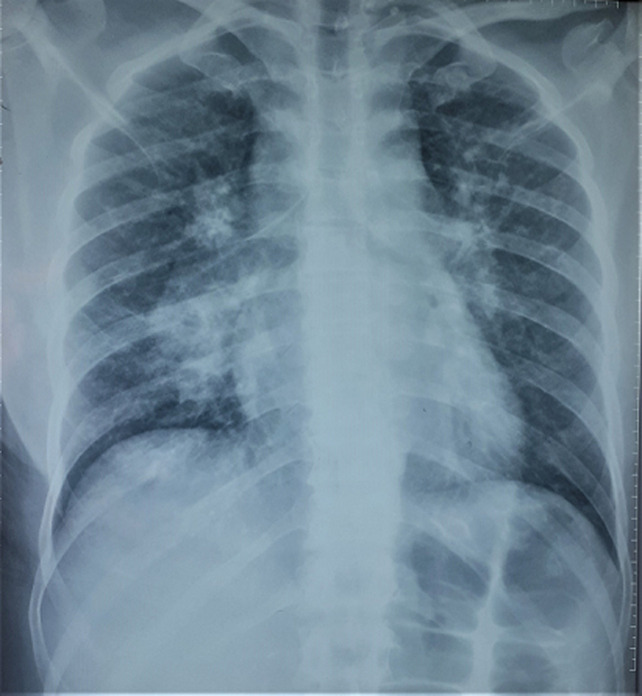
bilateral mediastino-pulmonary opacities associated with a diffuse micronodular infiltration

**Figure 2:**
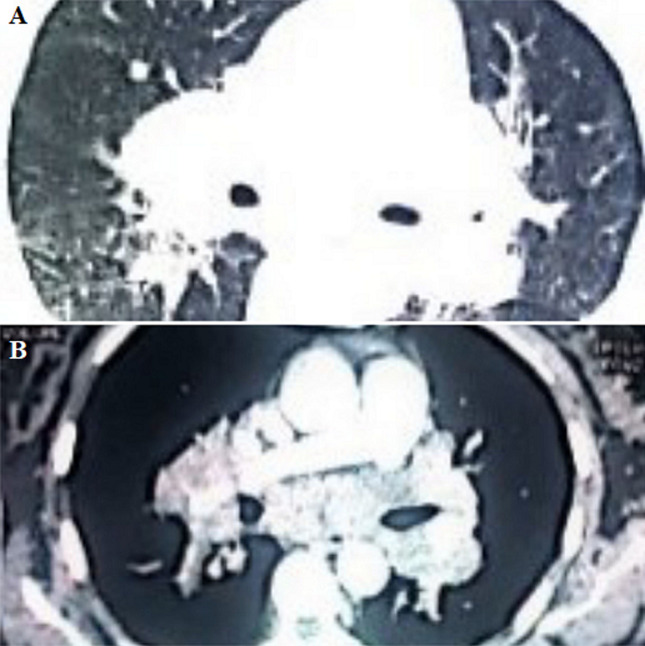
(A, B) chest CT axial section (medial and parenchymal window) bilateral and symmetrical non-compressive medial and para hilar adenopathy masses; bilateral para hilar peribronchovascular thickening; nodular thickening of the septal lines. Bilateral confluent basal sparse micronodules with Galaxie sign

**Figure 3:**
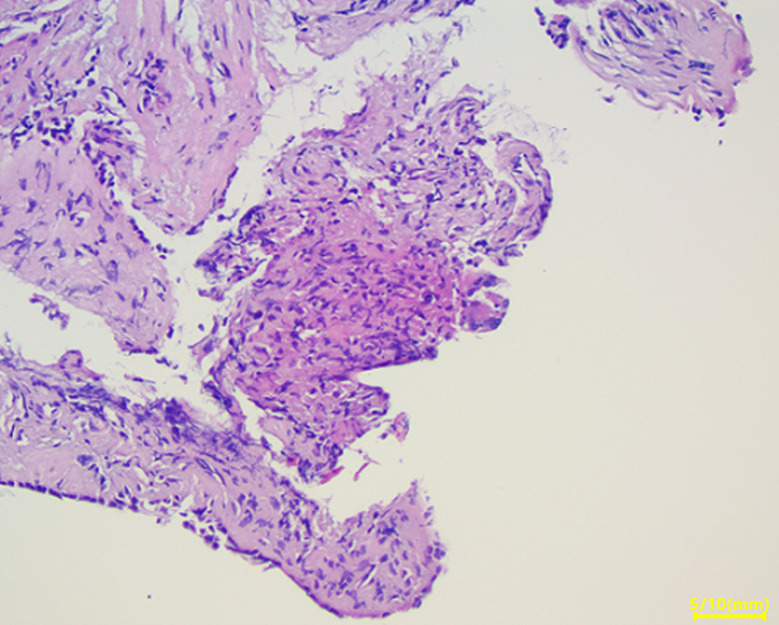
epithelial-giganto-cellular granuloma without caseous necrosis with no evidence of malignancy on bronchial spur biopsy

**Figure 4:**
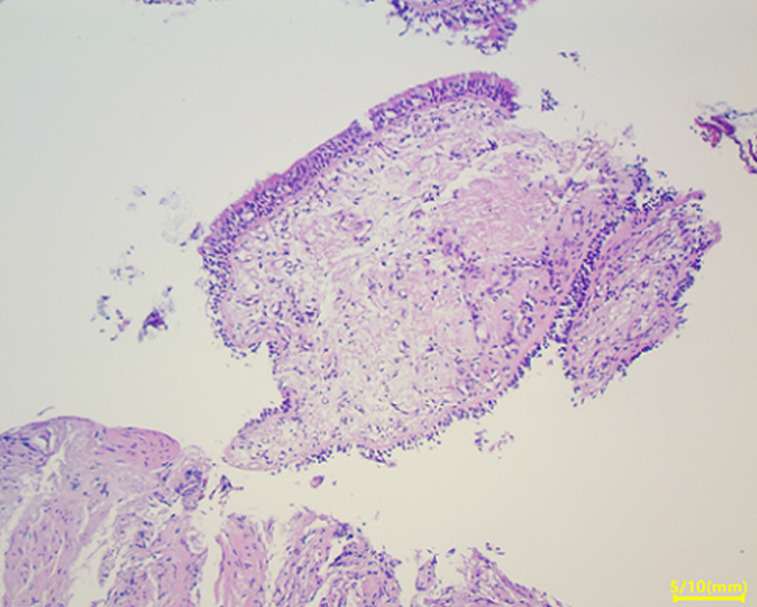
salivary parenchyma without abnormality with no sign of malignancy on biopsy of accessory salivary glands

**Clinical findings:** the clinical examination revealed a preserved general condition with a temperature of 38°C, symmetrical erythematous macules on the lower limbs in connection with erythema nodosum, left facial paralysis and bilateral sequelae of anterior uveitis on ophthalmologic examination. Chest X-ray showed bilateral mediastinopulmonary opacities associated with diffuse micronodular infiltration (Figure 1). The thoracic CT scan showed a magma of bilateral and symmetrical non-compressive medial and para hilar adenopathies, the largest of which measured 60 mm (right para hilar); bilateral para hilar peribronchovascular thickening; nodular thickening of the septal lines, with a pearly appearance of the scissures, more marked at the upper level; bilateral confluent basal sparse micronodules producing the sign of Galaxie (Figure 2). Cervical ultrasonography showed parotitis with swollen, hyper echogenic and heterogeneous glands with thick septa. The radio-clinical picture evoked a Lofgren's syndrome associated with a Heerfordt's syndrome. Further investigations were undertaken as part of a workup for sarcoidosis. Angiotensin-converting enzyme (ACE) was elevated to 104IU/L, phosphocalcic, urinary and serological tests were normal. Inflammation markers were elevated with lymphopenia on the blood count. The flexible bronchoscopy performed found a diffuse 1^st^ degree inflammatory state with no visible buds. A bronchial spur biopsy was performed, which revealed an epithelial-giganto-cellular granuloma without necrosis and no signs of malignancy (Figure 3). The bronchoalveolar lavage showed an inflammatory cytology rich in lymphocytes. Accessory salivary gland biopsy revealed salivary parenchyma without histopathological abnormalities and no evidence of malignancy (Figure 4). The pre-therapeutic work-up revealed normal blood glucose and viral serologies, an electrocardiogram and a transthoracic cardiac echography without abnormalities. Respiratory function tests showed hypoxemia with small airway involvement without carbon monoxide diffusion disorders.

**Therapeutic intervention:** a corticosteroid therapy 1mg/kg of Prednisolone was instituted with adjuvant measures to the corticosteroid therapy.

## Discussion

Sarcoidosis, also known as Besnier-Boeck-Shaumann disease (BBS), is a rare condition. It represents 5-10% of sarcoidosis and 4.4-5.6% as reported by Fukuhara *et al*. [6]. Genetically, the HLA-DRB1*04 allele appears to be protective against sarcoidosis and facilitates the occurrence of Heerfordt syndrome [7]. In our study, we reported a case of multi-systemic sarcoidosis in a 51-year-old female patient. This is in agreement with the literature which reveals a female predominance due to a hormonal factor with a second peak of the disease between 45 and 65 years of age corresponding to the peri-menopausal peak incidence [8].

Ocular manifestations are constant depending on the series, they are found in 25-60% of cases and are revealing of the disease in 10-20% of cases [9]. It is mainly anterior uveitis, which is quite characteristic as found in our study. All structures of the eyeball may be involved. It is sometimes revealing of the disease. It should be systematically investigated because of its functional prognosis, which requires rapid corticosteroid therapy to avoid sequelae. The involvement of the accessory salivary glands is present in 65% of the cases with a rare involvement of the main salivary glands sometimes resulting in bilateral parotitis (observed in 1-4% of cases) with xerostomia [4,6], as found in the series of Benjelloun H *et al*. [10]. In our study, the biopsy of the accessory salivary glands was unremarkable.

Neurological manifestations are rare. In our study, it resulted in facial paralysis which, depending on the series, is present in 25 to 50% of cases [4]. It is secondary to the involvement of the epineurium by sarcoid granulomas and to perineural inflammation [4,6]. The patient was treated with long-term corticosteroid therapy. It represents the first-line treatment of Heerfordt syndrome with a dose of 60 mg/day for 3 months followed by a degression of the corticosteroid therapy at a rate of 5 mg every 3 months [4,11].

## Conclusion

Heerfordt syndrome is a rare form of sarcoidosis. The diagnosis is simple in its complete form. The evolution is globally favorable under medical treatment with few functional sequelae. Nevertheless, the delay in diagnosis can jeopardize the functional prognosis, which can be responsible in some cases for blindness.
